# Proteins in stool as biomarkers for non‐invasive detection of colorectal adenomas with high risk of progression

**DOI:** 10.1002/path.5369

**Published:** 2020-01-13

**Authors:** Malgorzata A Komor, Linda JW Bosch, Veerle MH Coupé, Christian Rausch, Thang V Pham, Sander R Piersma, Sandra Mongera, Chris JJ Mulder, Evelien Dekker, Ernst J Kuipers, Mark A van de Wiel, Beatriz Carvalho, Remond JA Fijneman, Connie R Jimenez, Gerrit A Meijer, Meike de Wit

**Affiliations:** ^1^ Department of Pathology The Netherlands Cancer Institute Amsterdam The Netherlands; ^2^ Department of Medical Oncology Amsterdam UMC, VU University Medical Center Amsterdam The Netherlands; ^3^ Department of Epidemiology and Biostatistics Amsterdam UMC, VU University Medical Center Amsterdam The Netherlands; ^4^ Department of Pathology Amsterdam UMC, VU University Medical Center Amsterdam The Netherlands; ^5^ Department of Gastroenterology and Hepatology Amsterdam UMC, VU University Medical Center Amsterdam The Netherlands; ^6^ Department of Gastroenterology and Hepatology Amsterdam UMC, University of Amsterdam Amsterdam The Netherlands; ^7^ Department of Gastroenterology and Hepatology Erasmus MC University Medical Center Rotterdam The Netherlands

**Keywords:** colorectal cancer, high‐risk adenomas, biomarkers, early detection

## Abstract

Screening to detect colorectal cancer (CRC) in an early or premalignant state is an effective method to reduce CRC mortality rates. Current stool‐based screening tests, e.g. fecal immunochemical test (FIT), have a suboptimal sensitivity for colorectal adenomas and difficulty distinguishing adenomas at high risk of progressing to cancer from those at lower risk. We aimed to identify stool protein biomarker panels that can be used for the early detection of high‐risk adenomas and CRC. Proteomics data (LC–MS/MS) were collected on stool samples from adenoma (*n* = 71) and CRC patients (*n* = 81) as well as controls (*n* = 129). Colorectal adenoma tissue samples were characterized by low‐coverage whole‐genome sequencing to determine their risk of progression based on specific DNA copy number changes. Proteomics data were used for logistic regression modeling to establish protein biomarker panels. In total, 15 of the adenomas (15.8%) were defined as high risk of progressing to cancer. A protein panel, consisting of haptoglobin (Hp), LAMP1, SYNE2, and ANXA6, was identified for the detection of high‐risk adenomas (sensitivity of 53% at specificity of 95%). Two panels, one consisting of Hp and LRG1 and one of Hp, LRG1, RBP4, and FN1, were identified for high‐risk adenomas and CRCs detection (sensitivity of 66% and 62%, respectively, at specificity of 95%). Validation of Hp as a biomarker for high‐risk adenomas and CRCs was performed using an antibody‐based assay in FIT samples from a subset of individuals from the discovery series (*n* = 158) and an independent validation series (*n* = 795). Hp protein was significantly more abundant in high‐risk adenoma FIT samples compared to controls in the discovery (*p* = 0.036) and the validation series (*p* = 9e‐5). We conclude that Hp, LAMP1, SYNE2, LRG1, RBP4, FN1, and ANXA6 may be of value as stool biomarkers for early detection of high‐risk adenomas and CRCs. © 2019 Authors. *Journal of Pathology* published by John Wiley & Sons Ltd on behalf of Pathological Society of Great Britain and Ireland.

## Introduction

Colorectal cancer (CRC) remains a major health care problem, representing 6.1% of all cancers worldwide 1. Early detection through population screening is an efficient method to reduce the burden of CRC, and screening programs have been implemented in many countries 2. Screening programs aim to detect CRC at a curable stage or when it is still at a precursor non‐malignant stage (i.e. colorectal adenoma), and have been proven to reduce CRC mortality rates 3, 4, 5. Most population screening programs use a fecal immunochemical test (FIT) as a triage test to colonoscopy 2. In this setting, all participants with a positive FIT are referred for colonoscopy, during which adenomas and early cancers can be diagnosed and removed.

The reported sensitivity of FIT depends on the study characteristics but is overall high for CRC (67–86%) and relatively low for colorectal adenomas (29–35%), leaving room for improvement 6, 7, 8. It has been suggested that an increase in sensitivity for colorectal adenomas is the best approach to make CRC screening more cost‐effective and efficient 9, 10, 11. However, detecting all adenomas during screening is not the aim, as only approximately 5% of all adenomas are expected to develop into cancer 12. Advanced adenomas, defined as adenomas with a size of ≥ 10 mm, a villous component of ≥ 25%, and/or high‐grade dysplasia, are currently regarded as an intermediate endpoint for CRC in screening programs, since advanced adenomas are considered to carry a higher risk of developing into CRC than non‐advanced adenomas 13, 14, 15. Based on the fact that advanced adenomas are far more prevalent than CRC, not all advanced adenomas are expected to progress 12.Therefore, it is important to develop new screening tests directed at the identification of those lesions with the highest risk of progression.

Cancer is caused by DNA alterations, including specific changes in DNA copy numbers. Gains of chromosomal arms 8q, 13q, and 20q, and losses of 8p, 15q, 17p, and 18q have been associated with adenoma‐to‐carcinoma progression (i.e. cancer‐associated events or CAEs) 16, 17. Adenomas carrying two or more CAEs are considered at high risk of progression, i.e. high‐risk adenomas 17. Approximately 23–36% of advanced adenomas and 1.7–4.8% of non‐advanced adenomas were reported to be high‐risk adenomas 18. Based on the incidence of CRC, the molecularly defined high‐risk adenoma phenotype may better reflect the true progression risk than the advanced adenoma phenotype.

We have previously reported on stool protein biomarkers, which increased sensitivity compared with hemoglobin for detection of CRC and advanced adenomas 19. In contrast to the previous study where the focus was on advanced adenomas, here a molecularly‐defined intermediate endpoint was applied for biomarker discovery. In this study, we set out to further explore the same proteomics dataset for identification of protein biomarkers that are specifically suited for the detection of molecularly defined high‐risk adenomas.

## Materials and methods

**Figure 1:**
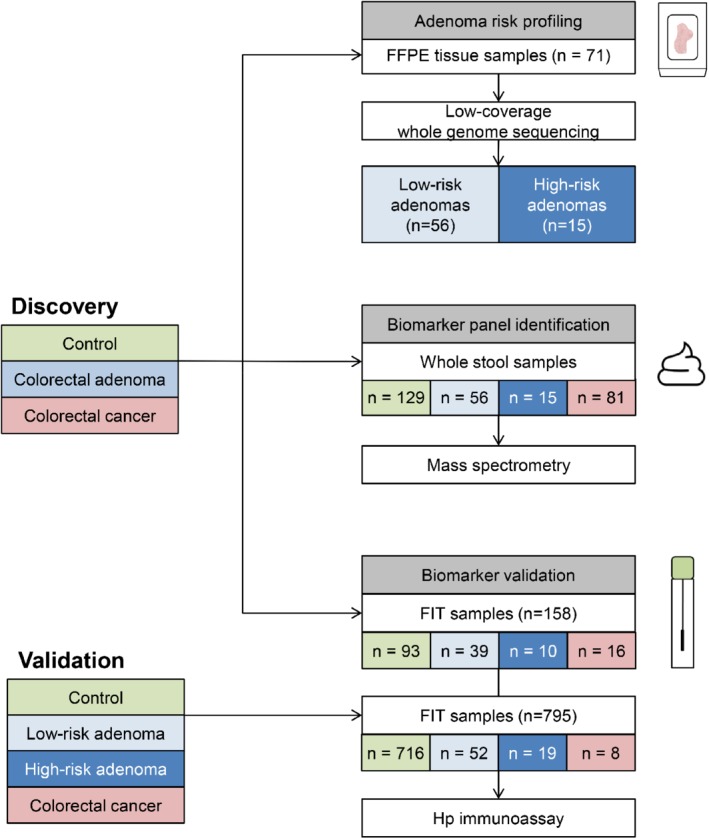
Overview of the design of this study. The discovery series consisted of control, colorectal adenoma, and colorectal cancer (CRC) samples. FFPE tissue blocks were obtained from 71 adenoma patients and low‐coverage whole‐genome sequencing was performed to identify DNA copy number aberrations. Fifteen high‐risk adenomas were identified according to their DNA copy number profiles. Whole stool samples of individuals from the discovery series were used for mass spectrometry proteomics analysis. Proteins identified were used for biomarker panel identification for high‐risk adenomas and high‐risk adenomas together with CRCs. An immunoassay was applied on 158 FIT samples from the discovery series and 795 FIT samples from the validation series for biomarker validation, to evaluate quantitative difference of Hp between controls, low‐risk adenomas, high‐risk adenomas, and CRCs.

The design of the study is presented in Figure 1.

### Samples

Informed consent was obtained from all subjects who provided stool and FIT samples. Collection, storage, and use of patient‐derived tissue and data were performed in compliance with the ‘Code for Proper Secondary Use of Human Tissue in The Netherlands’ by the Dutch Federation of Biomedical Scientific Societies 20.

### Stool, tissue, and FIT samples of the discovery series

For discovery, whole stool samples from 293 individuals diagnosed with CRC (*n* = 81), advanced adenoma (*n* = 40) or non‐advanced adenoma (*n* = 43) as most advanced lesion, and individuals without colorectal neoplasia (*n* = 129), further referred to as ‘controls’, were collected from a referral population that underwent colonoscopy at multiple centers in The Netherlands and Germany between 2005 and 2012. Sample description and processing have been previously described 19. In total for 71 adenoma patients, formalin‐fixed, paraffin embedded (FFPE) tissue samples were available and requested from the pathology archive of the Amsterdam UMC, location VUmc, The Netherlands. In total, 95 tissue samples were retrieved, as some individuals carried multiple adenomas.

From a subset of the individuals from the discovery series (*n* = 162), FIT samples (OC‐sensor; Eiken Chemical, Tokyo, Japan) were obtained prior to colonoscopy. These included patients diagnosed with CRC (*n* = 17), high‐risk adenoma (*n* = 10) or low‐risk adenomas (*n* = 39) as most advanced lesion, and controls (*n* = 96).

### FIT samples of the validation series

Between June 2009 and July 2010, in a population‐based screening study [COlonoscopy or COlonography for Screening (COCOS) trial] run in The Netherlands, asymptomatic individuals were invited for primary colonoscopy screening 21, 22. Screening participants allocated to the colonoscopy arm of the COCOS trial were invited to collect a FIT sample (OC‐sensor; Eiken Chemical) prior to their screening colonoscopy. FIT samples from 795 individuals diagnosed with CRC (*n* = 8), high‐risk adenomas (*n* = 19) or low‐risk adenomas (*n* = 52) as most advanced lesion, or without colorectal neoplasia (*n* = 716) were used for validation.

### DNA copy number analysis using low‐coverage whole‐genome sequencing

DNA was isolated from FFPE tissues with a column‐based method (QIamp DNA microkit; Qiagen, Hilden, Germany) as described before 18, 23. DNA copy number analysis (supplementary material, Supplementary materials and methods) and status for adenomas of the discovery and the validation series have been reported previously 18; data are available in the European Genome and Phenome Archive (EGAS0000100295). If two or more CAEs were present, an adenoma was classified as high‐risk adenoma 17, 18. Individuals with at least one high‐risk adenoma were defined as high risk.

### LC–MS/MS data analysis

The tandem mass spectrometry (LC–MS/MS) data on the stool samples of the 293 individuals were readily available and described previously (PRIDE ID: PXD007767) 19. Protein identification was performed with MaxQuant 24 as described previously 19 with some adaptations (see supplementary material, Supplementary materials and methods).

### Protein biomarker panel identification with logistic regression

An overview of the data analysis approach is presented in supplementary material, Figure S1. Proteins with higher abundance in cases (high‐risk adenomas or high‐risk adenomas and CRCs) compared with controls constituted input for selecting biomarker panels. Logistic regression analysis with Lasso regularization was used to identify biomarker panels consisting of two, three or four proteins that best distinguish cases from controls. A leave‐one‐out cross‐validation procedure was applied to evaluate the performance of the model. Cross‐validated logistic predictions were obtained. Receiver operating characteristic (ROC) analysis was used to evaluate the performance of protein panels to discriminate cases from controls by calculating the partial area under the curve (pAUC) between specificity of 95% and 100%, and by calculating sensitivity at 95% specificity. The pAUC was compared with the pAUC of haemoglobin (HBA1). *P* values were obtained with the stratified bootstrap resampling of case/control labels of the individuals with 2000 permutations 25.

### Haptoglobin (Hp) quantification in FIT samples

FIT samples from both the discovery and the validation series were analyzed with an antibody‐based assay (Figure 1). From the 162 FIT samples in the discovery series, four were excluded due to technical reasons (controls *n* = 3, CRC *n* = 1), leaving 158 samples for Hp quantification. Immunoassays for Hp employing a sandwich immunoassay format and electrochemiluminescence (ECL) detection were carried out on commercial instrumentation and multi‐well plate consumables from Meso Scale Diagnostics, LLC (MSD; Rockville, MD, USA); more details may be found in supplementary material, Supplementary materials and methods 26. All samples were analyzed in duplicate and final analyses were performed on mean concentrations.

### FIT values – correlation analysis

In the discovery series, hemoglobin (HBA1 and HBB) and haptoglobin (Hp) protein abundance as determined by mass spectrometry were compared with FIT values in the same samples. Missing values were excluded from the analysis. Spearman correlation analysis was performed on normalized spectral counts of HBA1, HBB, Hp and FIT values, correlation coefficients (rho) and *P* values were obtained.

## Results

### Characterization of cancer‐associated events in colorectal adenomas

In total, 95 adenomas from 71 adenoma patients from the discovery series were available for CAE identification as was described before (supplementary material, Figure S2) 18. A complete overview of the frequencies and the associations with adenoma histologic features may be found in supplementary material, Table S1. Two CAEs or more, indicating a higher risk of progression, were identified in 15.8% of all adenomas (*n* = 15; further referred to as high‐risk adenomas), in 36.4% (12/33) of advanced adenomas, and in 4.8% (3/62) of non‐advanced adenomas (supplementary material, Table S1 and Figure S2).

### Protein profiling and selection of candidate biomarkers

**Figure 2:**
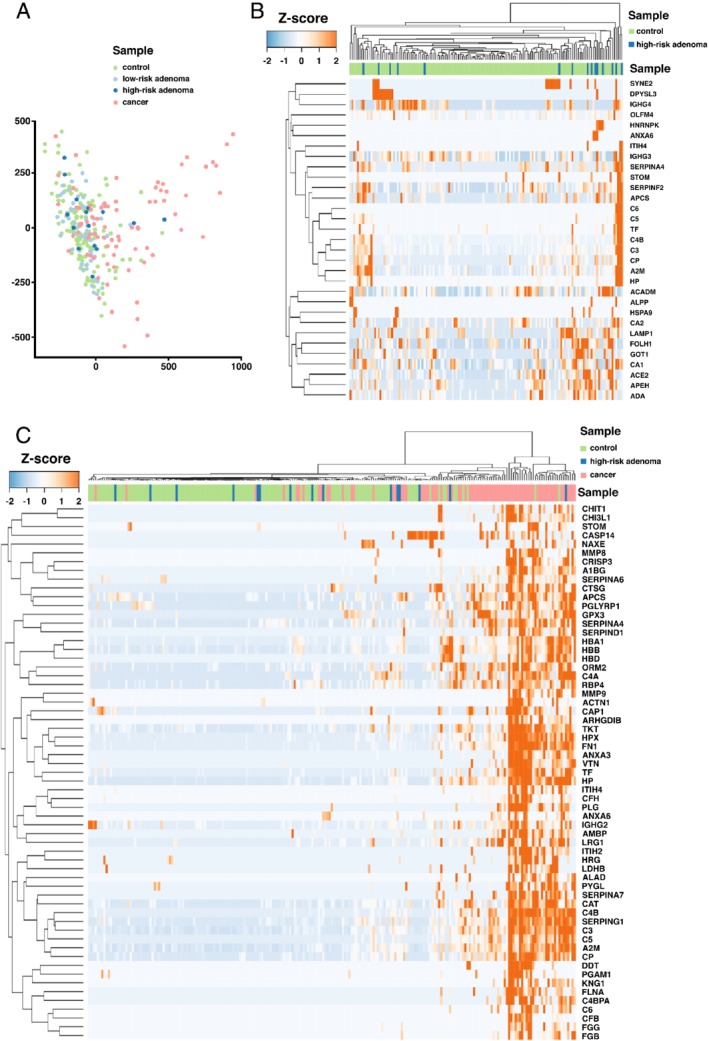
Proteomics profiling of human stool samples. (A) Multidimensional scaling of protein expression profiles of stool samples derived from controls (*n* = 129), individuals with low‐risk adenomas (*n* = 56), high‐risk adenomas (*n* = 15), and cancers (*n* = 79). (B) Hierarchical clustering of protein profiles of stool samples derived from high‐risk adenomas and controls based on 31 proteins expressed more highly in high‐risk adenomas compared with controls. (C) Hierarchical clustering of protein profiles of stool samples derived from CRCs, high‐risk adenomas, and controls based on 61 proteins expressed more highly in CRCs and high‐risk adenomas compared with controls.

In the discovery series, proteomics profiling of all stool samples revealed 792 protein groups (FDR ≤ 0.01; supplementary material, Table S2). Correlation analysis was performed between FIT values obtained from a subsample of the same bowel movement and normalized spectral counts for hemoglobin, in particular for HBA1 and HBB separately. Significant positive correlations were identified for both HBA1 (rho = 0.46, *p* < 0.001) and HBB (rho = 0.43, *p* < 0.001; supplementary material, Figure S3). Dimensionality reduction performed on the protein expression profiles distinguished stool samples from CRC patients from those with adenomas or controls (Figure 2A). To identify proteins that discriminate high‐risk adenomas from controls, we performed differential protein expression analysis. This yielded 31 proteins more abundant in high‐risk adenoma stool samples (log_2_ fold‐change > 0 and *p* ≤ 0.1; Figure 2B). Additionally, we performed differential protein expression analysis to identify proteins differentiating all screen‐relevant lesions, i.e. CRCs and high‐risk adenomas, from controls. Application of the same threshold revealed 125 protein groups to be more highly expressed in high‐risk adenomas and CRCs. For further analysis, a more stringent threshold was applied (i.e. log_2_ fold‐change ≥ 2, adjusted *p* ≤ 0.05) and revealed 61 proteins more abundant in screen‐relevant lesions compared with controls (Figure 2C). Significant overlap was identified between differentially expressed proteins from both analyses (*p* = 1.47e^−4^, hypergeometric test) with 13 proteins overlapping: CP, Hp, A2M, C3, C5, APCS, TF, ANXA6, C4B, C6, STOM, SERPINA4, and ITIH4.

### Biomarker panel selection for high‐risk adenomas

The proteomics dataset was further investigated to find biomarker panels of complementary proteins that would perform better than hemoglobin in distinguishing individuals with high‐risk adenomas from controls and a combination of high‐risk adenomas and CRCs from controls. Panels of two, three or four proteins were examined. To evaluate the diagnostic performance of each biomarker panel in the context of population screening, we compared its performance to hemoglobin, which is the protein currently used in CRC screening by means of FIT. Since FIT values were not available for the whole dataset, the performance of the biomarker panel was compared with HBA1 quantified by LC–MS/MS as a substitute (for comparison to FIT, see supplementary material, Figure S4). The analysis was done on a partial AUC (pAUC) at the specificity level between 95% and 100% and sensitivity was evaluated at 95% specificity, since high specificity is pivotal for the success of a population screening program.

**Figure 3:**
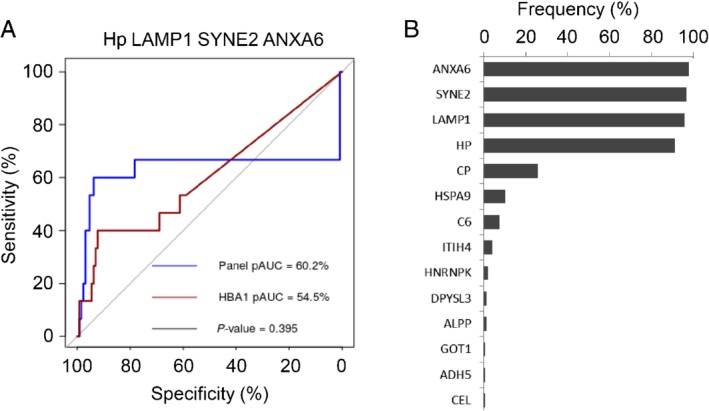
Biomarker panels from logistic regression analysis to identify high‐risk adenomas and CRCs. (A) ROC curve of the regression model using the four‐biomarker panel (Hp, LAMP1, SYNE2, and ANXA6) to distinguish between stool samples from individuals with high‐risk adenomas (*n* = 15) and controls (*n* = 129). ROC curve was obtained from logistic regression predictions from the leave‐one‐out cross‐validation analysis. Partial area under the curve (pAUC) was calculated for specificity of 95–100% and compared with pAUC of hemoglobin to obtain the *P* value. (B) Frequency plot of biomarkers occurring in the regression models built during the cross‐validation analysis to distinguish between the high‐risk adenomas and controls. Four proteins were clearly selected more frequently by the Lasso regularization in the cross‐validation analysis.

First, we applied logistic regression with Lasso regularization on the 31 up‐regulated proteins in high‐risk adenomas to identify a biomarker panel (see supplementary material, Figure S1 for the data analysis overview). In the resulting regression model, Hp, LAMP1, SYNE2, and ANXA6 were selected, while the models for three or two proteins were not built, as due to the Lasso regularization the coefficients for LAMP1, SYNE2, and ANXA6 shrunk to zero at the same time, meaning that the three proteins were excluded from the regression model at once. Then the performance of the model was evaluated using leave‐one‐out cross‐validation and an ROC analysis was used to compare to the performance of hemoglobin. In the cross‐validation procedure, only models based on four proteins were included (Figure 3). Despite the fact that the pAUC of the biomarker panel (pAUC = 60.2%) was higher than that for HBA1 (pAUC = 54.5%), the difference was not significant. At the specificity level of 95%, the biomarker panel could identify 8 out of 15 high‐risk adenomas (sensitivity = 54%, CI = [27, 79%]), which was more than hemoglobin (sensitivity = 13%, CI = [2, 40%], see Table 1A). The markers most frequently selected in the cross‐validation procedure were Hp, LAMP1, SYNE2, and ANXA6, with a frequency of over 90%, indicating that these proteins have the most discriminative roles in the regression models (Figure 3B).

**Table 1:** Confusion matrix for the cross‐validated performance of the models of biomarker panels. Performance of the biomarker panel regression models was evaluated at 95% specificity and compared with hemoglobin. (A) High‐risk adenomas versus controls and (B) high‐risk adenomas and CRCs versus controls

A			
Protein(s)	Control	High‐risk adenoma	Sensitivity at 95% specificity [95% confidence intervals]
Hp, LAMP1, SYNE2, ANXA6			
Predicted control	123	7	53% [27–79%]
Predicted high‐risk adenoma	6	8	
HBA1			
Predicted control	123	13	13% [2–40%]
Predicted high‐risk adenoma	6	2	

B			
Protein(s)	Control	High‐risk adenoma or CRC	Sensitivity at 95% specificity [95% confidence intervals]
Hp, LRG1, RBP4, FN1			
Predicted control	123	36	62% [51–72%]
Predicted high‐risk adenoma or CRC	6	58	
Hp, LRG1			
Predicted control	123	32	66% [55–75%]
Predicted high‐risk adenoma or CRC	6	62	
HBA1			
Predicted control	123	56	40% [30–51%]
Predicted high‐risk adenoma or CRC	6	38	

The model was also applied to low‐risk adenomas. Here, five (9%, CI = [3, 20%]) low‐risk adenomas were classified as cases and 51 (91%) as controls, indicating that this biomarker panel has a high specificity for the identification of high‐risk adenomas (see supplementary material, Table S3).

### Biomarker panel selection for high‐risk adenomas and CRCs combined

**Figure 4:**
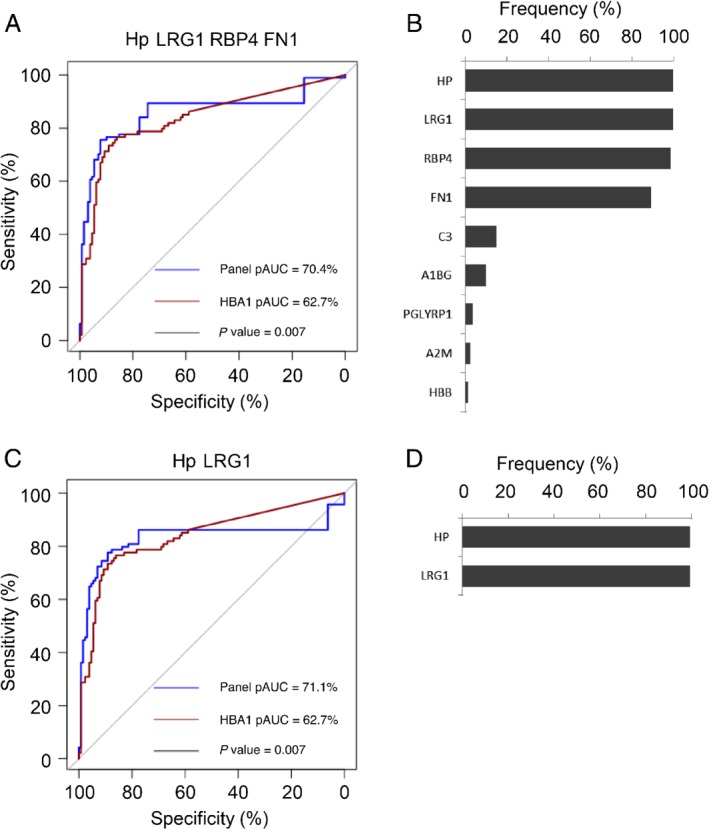
Biomarker panels from logistic regression analysis to identify high‐risk adenomas and CRCs. (A) ROC curve of the model based on the panel of four biomarkers (Hp, LRG1, RBP4, and FN1) for high‐risk adenomas and CRCs (*n* = 94) compared with controls (*n* = 129). ROC curve was obtained from logistic regression predictions from the leave‐one‐out cross‐validation analysis. (B) Frequency plot of biomarkers occurring in the regression models built during the cross‐validation analysis to discriminate high‐risk adenomas and CRCs from controls based on four proteins. Four proteins were clearly selected more frequently by the Lasso regularization in the cross‐validation analysis. (C) ROC curve of the model based on the panel of two biomarkers (Hp and LRG1) for high‐risk adenomas and CRCs (*n* = 94) compared with controls (*n* = 129). ROC curve was obtained from logistic regression predictions from the leave‐one‐out cross‐validation analysis. (D) Frequency plot of biomarkers occurring in the regression models built during the cross‐validation analysis to discriminate high‐risk adenomas and CRCs from controls based on two proteins. The same two proteins were consistently selected in the cross‐validation analysis.

Next, we performed the same analysis for the 61 up‐regulated proteins in stool samples derived from individuals with high‐risk adenomas and CRCs. The model with four protein biomarkers consisted of Hp, LRG1, RBP4, and FN1; the model with three features was not built, as due to Lasso regularization the coefficients of FN1 and RBP4 shrunk to zero at the same time; and the model of two proteins consisted of Hp and LRG1. In the cross‐validation procedure, the models of four and two proteins were evaluated (Figure 4). The cross‐validated pAUCs of the four‐ (pAUC = 70.4%) and two‐protein (pAUC = 71.1%) models significantly outperformed hemoglobin (pAUC HBA1 = 62.7%, both *p* = 0.007; Figure 4A,C). At the specificity level of 95%, the four‐ and two‐biomarker panels could identify 58 and 62 out of 94 cases, respectively (sensitivity = 62 and 66%, CI = [51, 72%] and [55, 75%]), which was more than HBA1 (sensitivity = 40%, CI = [30, 51%]; Table 1B). The most frequent proteins included in the four‐protein regression models in the cross‐validation procedure were Hp, LRG1, RBP4, and FN1, with frequencies of over 90%, confirming their predictive characteristics and the stability of the model (Figure 4B). The model with two proteins always consisted of Hp and LRG1 in the cross‐validation procedure, indicating their strongest predictive characteristics (Figure 4D).

The four‐ and two‐protein models were also tested for identification of low‐risk adenomas. The four‐protein panel classified six (11%, CI = [4, 22%]) out of 56 low‐risk adenomas as cases and 50 (89%) as controls, while the two‐protein panel classified seven (13%, CI = [5, 24%]) low‐risk adenomas as cases and 49 (87%) as controls (supplementary material, Table S3).

When focusing on the overlap of up‐regulated proteins in both comparisons and the biomarker panels selected by Lasso regularization, Hp was the only protein present in all panels. This suggests that Hp might be a crucial component when distinguishing between high‐risk adenomas and CRCs from controls.

### Validation of Hp expression by immunoassay in FIT samples

As Hp forms a complex with hemoglobin, we explored if the protein abundance as measured by mass spectrometry was correlated to FIT and/or hemoglobin (supplementary material, Figure S3). As expected, we observed a strong correlation to HBA1 and HBB and a somewhat weaker correlation to FIT (correlation coefficient 0.77, 0.67, and 0.55, respectively; *p* < 0.001 for all comparisons). In line with this, Hp as a single marker did not outperform FIT (supplementary material, Figure S5).

**Figure 5:**
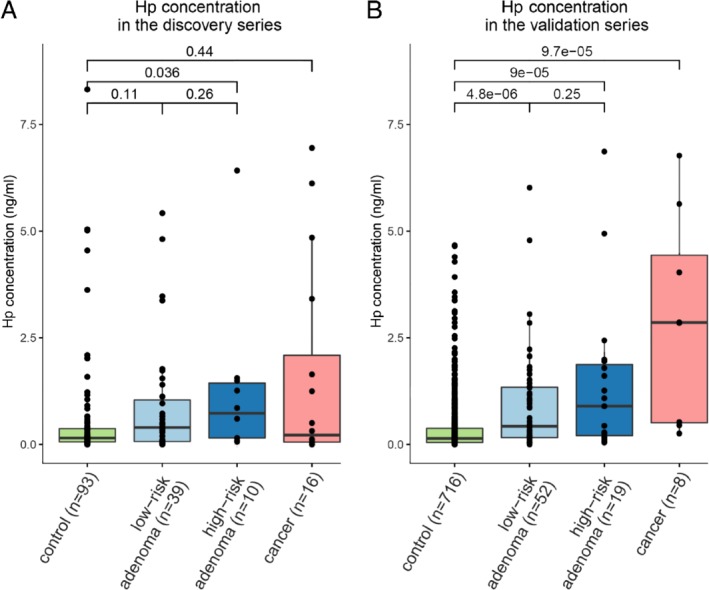
Validation of Hp protein expression with the use of an immunoassay. (A) The discovery series. (B) The validation series.

Nevertheless, as in the regression models Hp was consistently selected in all three marker panels, we further explored the Hp levels in two FIT cohorts. Using an immunoassay, Hp quantification was successfully performed in FIT samples of a subset of individuals from the discovery series (*n* = 158; 16 CRCs, 10 high‐risk adenomas, 39 low‐risk adenomas, and 93 controls). A significantly higher concentration of Hp was identified in the high‐risk adenoma samples compared with the controls (fold‐change = 1.9, *p* = 0.036; Figure 5A). Additionally, an independent validation series was used (Figure 5B), which consisted of 716 controls, 52 low‐risk adenomas, 19 high‐risk adenomas, and 8 CRCs. Here, a higher abundance of Hp in high‐risk adenomas (fold‐change =15.9, *p* = 9e^−5^) and CRCs (fold‐change = 42.6, *p* = 9.7e^−5^) compared with controls was confirmed. This confirms our findings by mass spectrometry and suggests that Hp can be applied as a biomarker for high‐risk adenomas and CRCs.

## Discussion

It is well known that not all colorectal adenomas will progress to CRC. This underlines the importance of developing screening tests for the detection of specifically those adenomas that are at high risk of progressing to malignancy 27. The widely used FIT is not optimal for detecting such adenomas, and therefore additional biomarkers could aid in improving sensitivity for early detection of CRC. Proteins are an attractive category of molecules to be used as biomarkers for application in stool‐based CRC screening, as they can be measured in small sample volumes with simple economic assays like FIT 28. In the present study, we aimed to identify combinations of specific stool‐based protein biomarkers that outperform hemoglobin in the detection of molecularly defined high‐risk adenomas and CRCs. Based on their DNA copy number profiles, adenomas were classified into lesions at low or high risk of progressing to cancer 16, 17, 18. High‐risk adenomas comprised 15.8% of all adenomas and 36.4% of the advanced adenomas. Using mass spectrometry proteomics on stool samples and regression modeling, we selected marker panels consisting of up to four proteins that distinguish screen‐relevant lesions, i.e. high‐risk adenomas and CRCs, from controls. We identified a biomarker panel of Hp, LAMP1, SYNE2, and ANXA6 for identification of high‐risk adenomas and two biomarker panels – Hp and LRG1, as well as Hp, LRG1, RBP4, and FN1 – for identification of high‐risk adenomas and CRCs that outperformed hemoglobin. Since Hp was the single protein present in all three biomarker panels, it was selected for further validation. To test its applicability in a screening setting, we used antibody‐based assays on FIT samples for the validation experiments. The higher concentration of Hp in high‐risk adenomas and CRCs compared with controls was confirmed using an immunoassay in FIT samples of both the discovery series and a validation series.

Using mass spectrometry analysis of stool samples, we previously established protein panels that showed a higher sensitivity for advanced adenoma and CRC samples compared with hemoglobin 19. In the present study, we performed subsequent statistical analyses to select alternative candidate biomarkers, including the most promising protein combinations that may improve the current stool‐based CRC population screening in the detection of high‐risk adenomas and CRCs. Statistical analysis of discovery mass spectrometry proteomics datasets on complex samples like stool are challenging due to missing data. Therefore, two feature selection methods were used to select the best biomarker panels for identification of cases versus controls, accounting for the complexity of our dataset: the beta‐binomial test 29 and Lasso regularization in the regression modeling 30. The beta‐binomial test was used for detection of proteins more highly expressed in the cases than in the controls, while logistic regression with Lasso regularization was applied to select for the best combination of these more highly expressed proteins to distinguish cases from the controls. Lasso regularization shrinks coefficients of less importance or correlating features to zero, therefore achieving a sparser solution, i.e. a smaller number of features in the final regression model. This method not only avoids overfitting but also performs feature selection of the best performing model.

A limitation of this study was the small number of molecularly defined high‐risk adenoma patients (*n* = 15), which affected the performance of the model built on only high‐risk adenomas as cases. Based on our previous work, it was anticipated that only a limited number of even the morphologically defined advanced adenomas would carry two or more CAEs 18. However, the most relevant screening targets are CRCs as well as adenomas considered at high risk of progression. In line with this approach, combining CRCs and molecularly defined high‐risk adenomas increased the size of the set of cases, and improved the performance of the models. Moreover, in the discovery series, FIT results were not available for all samples (162 out of 277 samples), which limited the possibilities of direct comparison of the marker panels with FIT performance, especially for the high‐risk adenomas (*n* = 10 with FIT available).

The marker panels in the discovery phase consistently contained haptoglobin (Hp), which as the hemoglobin–haptoglobin complex has been previously investigated as a biomarker for CRC 31. The Hp–Hb complex has been suggested to render a more stable biomarker than Hb or Hp alone, and could therefore increase sensitivity for the more proximal lesions in the bowel 32. This, however, was not confirmed in the current study (data not shown). It has been described that the sensitivity for CRC does not increase with the detection of an Hp–Hb complex compared with hemoglobin alone, but the sensitivity for adenomas does 33. In this study, the sensitivity of the complex versus the single proteins could not be assessed. Nevertheless, using an antibody‐based assay, a higher abundance of Hp was confirmed in FIT samples of patients with high‐risk adenomas and CRCs in the discovery series and in a much larger independent validation series. These findings underline the importance of Hp as a biomarker for screen‐relevant lesions and hold promise for future application of Hp in CRC screening. Meanwhile, hemoglobin (HBA1, HBB or HBD) was not significantly differential between high‐risk adenomas and controls, and subsequently it was not selected in any of the biomarker panels, which is in line with the limited sensitivity of FIT for adenomas. Although one would expect that Hp is a marker of blood in the stool and therefore should not have complementary value to hemoglobin, our data suggest that Hp is of added value for the detection of high‐risk adenomas. A possible explanation may be that the Hp protein detected in stool is not only derived from blood but may also be derived from the CRC or high‐risk adenoma tissues. In line with this, Hp has been described to be expressed by colorectal cancer cells: both cell lines and within the tumor, where its expression was associated with the stage of progression 34.

Next to Hp, LAMP1, SYNE2, and ANXA6 were selected in the analysis for high‐risk adenomas, and also LRG1, RBP4, and FN1 for the high‐risk adenomas and CRCs. LAMP1 is a lysosome‐associated membrane protein which has been implicated in several tumor‐promoting activities such as promotion of metastasis, drug resistance, and cancer cell survival 35. The gene coding for LAMP1 is located on chromosome 13q, gain of which is one of the seven CAEs used for classifying adenomas as high‐risk. SYNE2 (or nesprin 2) is a nuclear envelope protein that is involved in the regulation of nuclear trafficking; even though its role in cancer is yet to be established, there are indications that its presence is pivotal in the DNA damage response 36. Since high‐risk adenomas are characterized by chromosomal gains and losses, the up‐regulation of SYNE2 might be linked to these DNA aberrations. ANXA6 is present at the cell membrane and in the endosomal compartments, where it functions as a multifunctional scaffolding protein. In that position, ANXA6 can contribute to many different processes including cancer cell migration and invasion 37. RBP4 has been linked to insulin resistance and has been shown to be present in the serum of breast cancer patients 38; it was previously described as a potential marker for colorectal advanced adenomas in stool 19. FN1 is an extracellular matrix protein that is involved in cell adhesion and migration processes; it has been shown to be present in the serum of patients with hepatocellular carcinoma and has been suggested as a biomarker for this disease 39. Finally, LRG1 has been reported to be highly up‐regulated in CRC, both at the mRNA and at the protein level 40, 41. An evident role in tumor development has been established for LRG1, as it stimulates proliferation and inhibition of apoptosis through regulating RUNX1 expression 40, 42. In addition, the protein is secreted and may therefore end up in blood or stool. Indeed, increased protein levels of LRG1 in plasma have been reported for colorectal cancer and colon adenoma patients 40, 43, 44. Altogether, for the majority of these biomarker proteins their potential involvement in tumor biology has been demonstrated. Further investigation is needed to evaluate the diagnostic potential of these protein biomarkers in a CRC screening setting.

The present study is unique because a molecularly‐defined intermediate endpoint was used for biomarker discovery, by applying chromosomal copy number alterations highly associated with colorectal adenoma‐to‐carcinoma progression. This is in contrast to the morphological features traditionally used to define the advanced adenoma intermediate endpoint. Our study resulted in the identification of novel protein biomarker panels with higher sensitivities for high‐risk adenomas and CRCs than HBA1, which have plausible roles in colorectal carcinogenesis. FIT has a low sensitivity for colon adenomas; by increasing the sensitivity for high‐risk adenomas, we can raise the detection rates for these lesions. Therefore, these biomarkers have the potential to improve current FIT‐based screening strategies.

## Author contributions statement

MAK, LJWB, VMHC, MAW, BC, RJAF, CRJ, GAM, and MdW conceived and designed the study. LJWB, SRP, SM, and MdW collected the data. MAK, LJWB, VMHC, CR, TVP, MAW, BC, RJAF, CRJ, GAM, and MdW performed analysis and interpretation of the data. MAK and MdW drafted the article. BC, RJAF, CRJ, GAM, and MdW obtained the funding. CJJM, ED, EJK, and GAM provided study material. MAK, VMHC, TVP, and MAW contributed to the statistical analysis. All authors made revisions of the manuscript for important intellectual content and had final approval of the submitted and published versions.

## Supporting information


**Supplementary materials and methods**

**Figure S1.** Overview of the data analysis approach for the biomarker panel identification
**Figure S2.** Frequency plots of DNA copy number aberrations in the adenomas
**Figure S3.** Spearman correlation analysis of hemoglobin (HBA1, HBB) and haptoglobin (HP) spectral counts and FIT values
**Figure S4.** Comparison of the biomarker panels to FIT values
**Figure S5.** Comparison of the diagnostic performance of FIT and haptoglobin (Hp) measured with an antibody‐based assay for high‐risk adenomas (A, B) and high‐risk adenomas with CRCs (C, D)

**Table S1.** Frequencies of cancer‐associated events and histologic features of the adenomas
**Table S2.** Overview of the proteomics data from the discovery series
**Table S3.** Performance of the biomarker panels in the dataset including low‐risk adenomas at the specificity level of 95%
