# Supplementary material for: Proteins in stool as biomarkers for non‐invasive detection of colorectal adenomas with high risk of progression
Source: J Pathol. 2020 Jan 13;250(3):288–98. doi: 10.1002/path.5369 (PMC7065084; doi:10.1002/path.5369)
Supplement: Supplementary file 1 — Supplementary materials and methods [file PATH-250-288-s001.docx]

**Proteins in stool as biomarkers for non-invasive detection of colorectal adenomas with high risk of progression**Komor *et al. J Pathol* DOI: 10.1002/path.5369

# Supplementary materials and methods

Reference numbers refer to the main text list

### DNA copy number analysis

In brief, isolated DNA was subjected to low-coverage whole-genome sequencing on a HiSeq 2000 (Illumina, San Diego, USA) in a 50-bp single-read modus using the Illumina Truseq Nano kit. Raw sequence reads were mapped to the human reference genome build GRCh37/hg19 and data were further analyzed using QDNAseq, CGHcall, CGHregions [18]. Adenomas were characterized for gains of chromosomal arms 8q, 13q, and 20q, and losses of 8p, 15q, 17p, and 18q.

### LC–MS/MS data analysis

In brief, Swissprot human reference FASTA file was used as the database (canonical and isoforms, obtained in October 2017, 20 237 entries). Contaminants and reversed proteins were removed. Protein groups with a positive Andromeda score were extracted. Proteins were quantified by spectral counting [45]. Protein groups were excluded from further analysis if they had missing data for over 15% of the cases, i.e. 13 samples for high-risk adenomas or 80 samples for high-risk adenomas and CRCs. Euclidean distance between samples was calculated based on their protein expression profiles and proteomics data were visualized using the multidimensional scaling algorithm. Differential protein expression analysis was performed using the beta-binominal test [29]; log_2_ fold-changes and *P* values were obtained. *P* values adjusted for multiple hypothesis testing were obtained with the Benjamini–Hochberg correction. Differential analysis was performed for the following groups and the following thresholds were applied to select for proteins more highly expressed in cases than in controls: stool samples from high-risk adenoma patients compared with samples from controls (log_2_ fold-change > 0 and *p* ≤ 0.1), and stool samples from CRC and high-risk adenoma patients compared with samples from controls (log_2_ fold-change ≥ 2 and adjusted *p* ≤ 0.05). Clustering of the proteins more highly expressed in cases than in controls was performed using hierarchical clustering, where protein abundances were normalized to *Z* scores. Subsequently, the Euclidean distance was used with ward linkage for samples and complete linkage for proteins.

### Haptoglobin quantification in FIT samples

The immunoassay for Hp employed a sandwich immunoassay format and electrochemiluminescence (ECL) detection was carried out on commercial instrumentation and multi-well plate consumables from Meso Scale Diagnostics, LLC (MSD; Rockville, MD, USA) [26]. The assay was run in MSD’s U-PLEX format. The U-PLEX format employs 96-well plates, in which each well comprises a screen-printed carbon ink electrode coated with a generic 10-plex array of binding reagents.

The capture antibodies (goat polyclonal; catalog number F21YF; MSD) were biotinylated with Sulfo-NHS-LC-Biotin (Thermo Fisher Scientific, Waltham, MA, USA) and coupled to U-PLEX linkers via biotin–streptavidin binding. Detection antibodies (goat polyclonal; catalog number F21YF; MSD) were conjugated to the MSD SULFO-TAG ECL label. The assay was run according to the following protocol using commercial diluents from MSD: (i) Capture antibody–linker conjugate, specific for the target, was prepared and used immediately or stored at 4 °C. To each well of the U-PLEX plate, 50 µl of this was added. The plates were incubated for 1 h at room temperature with shaking to allow the antibody arrays to assemble and then washed with 1X MSD Wash Buffer to remove excess unbound capture antibody. (ii) MSD Diluent 100 (99 µl) was combined with 1 µl of sample in each well of the plate, and the plates were incubated for 1 h at room temperature with shaking to bind Hp in the sample to the capture antibody in the well. Each plate was calibrated with an eight-point standard curve of purified Hp (50 µl per well) prepared in the same diluent; all samples were run in duplicate. (iii) After washing the wells to remove the unbound sample, 25 µl of SULFO-TAG-labeled detection antibody (in MSD Diluent 100) was added and incubated for an additional hour at room temperature with shaking to complete the immunoassay sandwich. (iv) Plates were washed to remove the unbound detection antibody and then the wells were filled with 150 µl of 2X MSD Read Buffer T with surfactant. ECL was measured on an MSD SECTOR Imager 6000 plate reader. The plate reader applies a voltage to the electrodes in each well and measures the light emission from each array spot.

The relationship of ECL signal to calibrator concentration was fitted to a four-parameter logistic (4-PL) model with 1/Y2 weighting. Concentrations for the test samples were calculated by back-fitting ECL signals to the 4-PL fit for each plate [26].
