# Supplementary material for: Proteins in stool as biomarkers for non‐invasive detection of colorectal adenomas with high risk of progression
Source: J Pathol. 2020 Jan 13;250(3):288–98. doi: 10.1002/path.5369 (PMC7065084; doi:10.1002/path.5369)
Supplement: Supplementary file 2 — Figure S1. Overview of the data analysis approach for the biomarker panel identification Figure S2. Frequency plots of DNA copy number aberrations in the adenomas Figure S3. Spearman correlation analysis of hemoglobin (HBA1, HBB) and haptoglobin (HP) spectral counts and FIT values Figure S4. Comparison of the biomarker panels to FIT values Figure S5. Comparison of the diagnostic performance of FIT and haptoglobin (Hp) measured with an antibody‐based assay for high‐risk adenomas (A, B) and high‐risk adenomas with CRCs (C, D) [file PATH-250-288-s002.docx]

**Proteins in stool as biomarkers for non-invasive detection of colorectal adenomas with high risk of progression**Komor *et al. J Pathol* DOI: 10.1002/path.5369


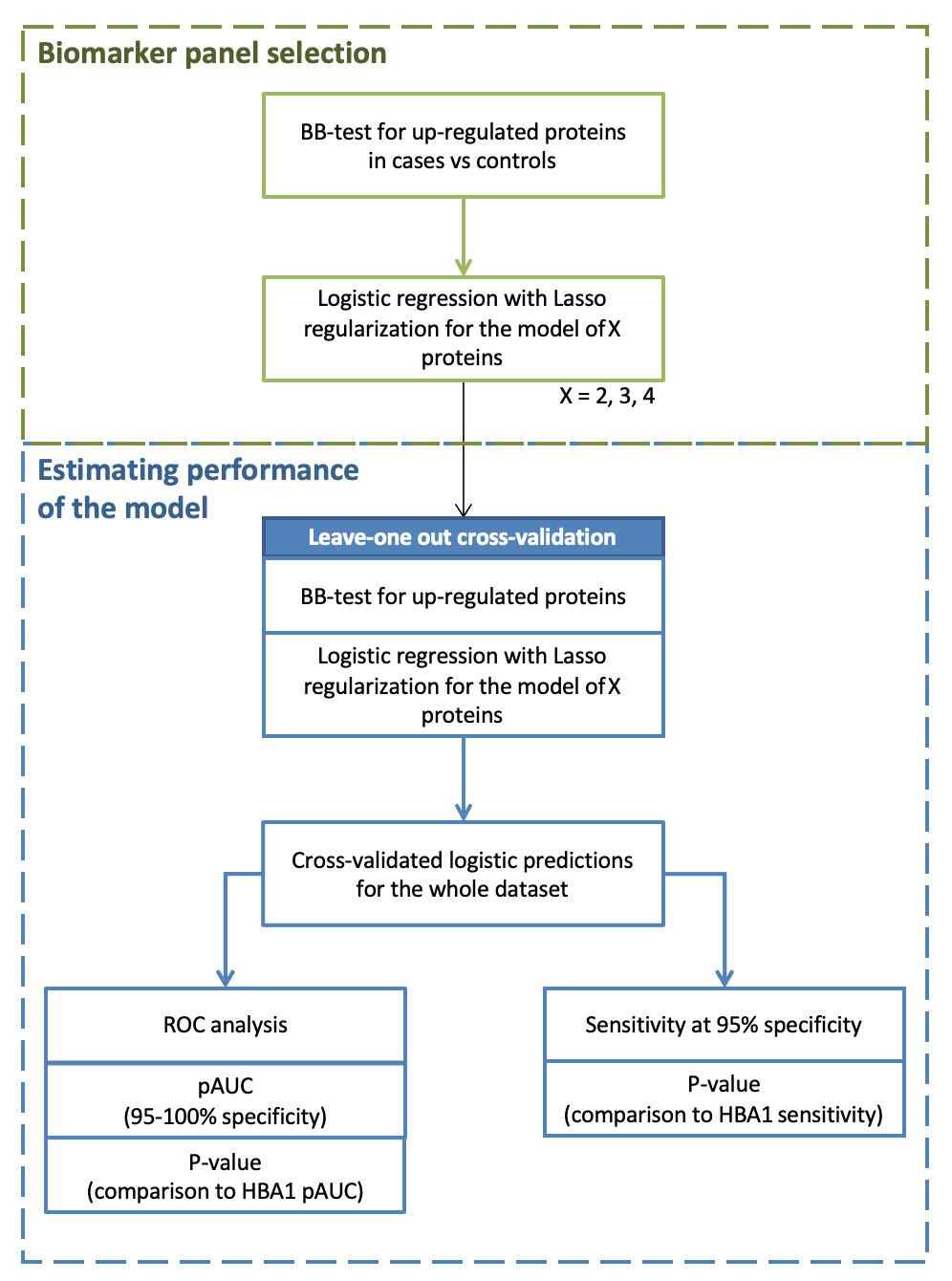


**Figure S1.** Overview of the data analysis approach for the biomarker panel identification. Feature selection was performed using the beta-binomial test (BB-test) in the comparative setting cases versus controls, in particular high-risk adenomas versus healthy controls and high-risk adenomas with CRCs versus healthy controls. Up-regulated proteins were selected using different thresholds for each comparison (see Materials and methods). Logistic regression with Lasso regularization was applied to build a model based on *X* features (where *X* is either two, three or four features). The performance of the model was evaluated using leave-one-out cross-validation, where feature selection with BB-test and logistic regression with Lasso regularization were repeated. Cross-validated performance of the built models was evaluated with respect to hemoglobin (HBA1) at high specificity levels.


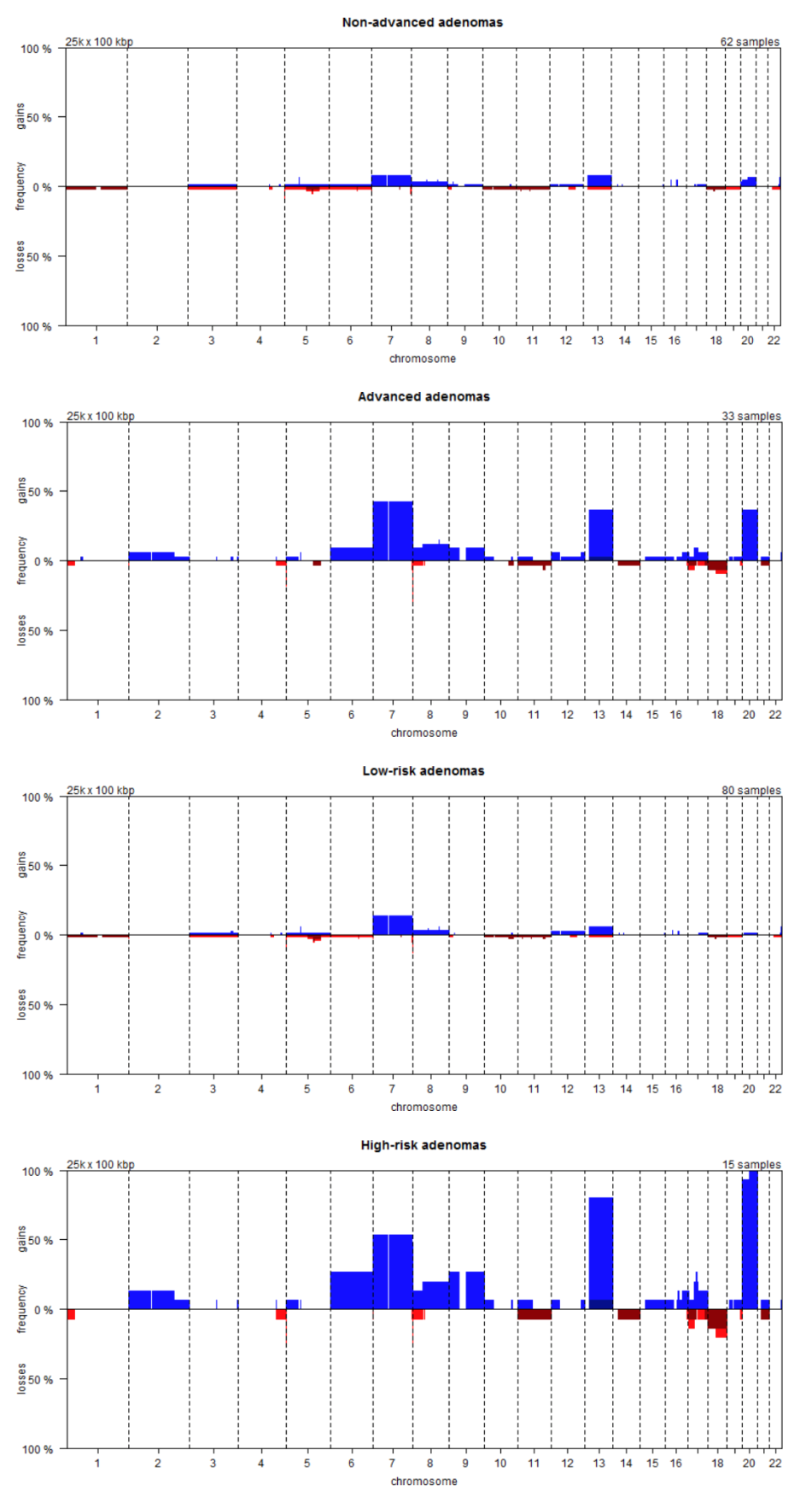


**Figure S2.** Frequency plots of DNA copy number aberrations in the adenomas. Copy number aberrations are plotted per set; in non-advanced adenomas (*n* = 62), advanced adenomas (*n* = 33), low-risk adenomas (*n* = 80), and high-risk adenomas (*n* = 15).


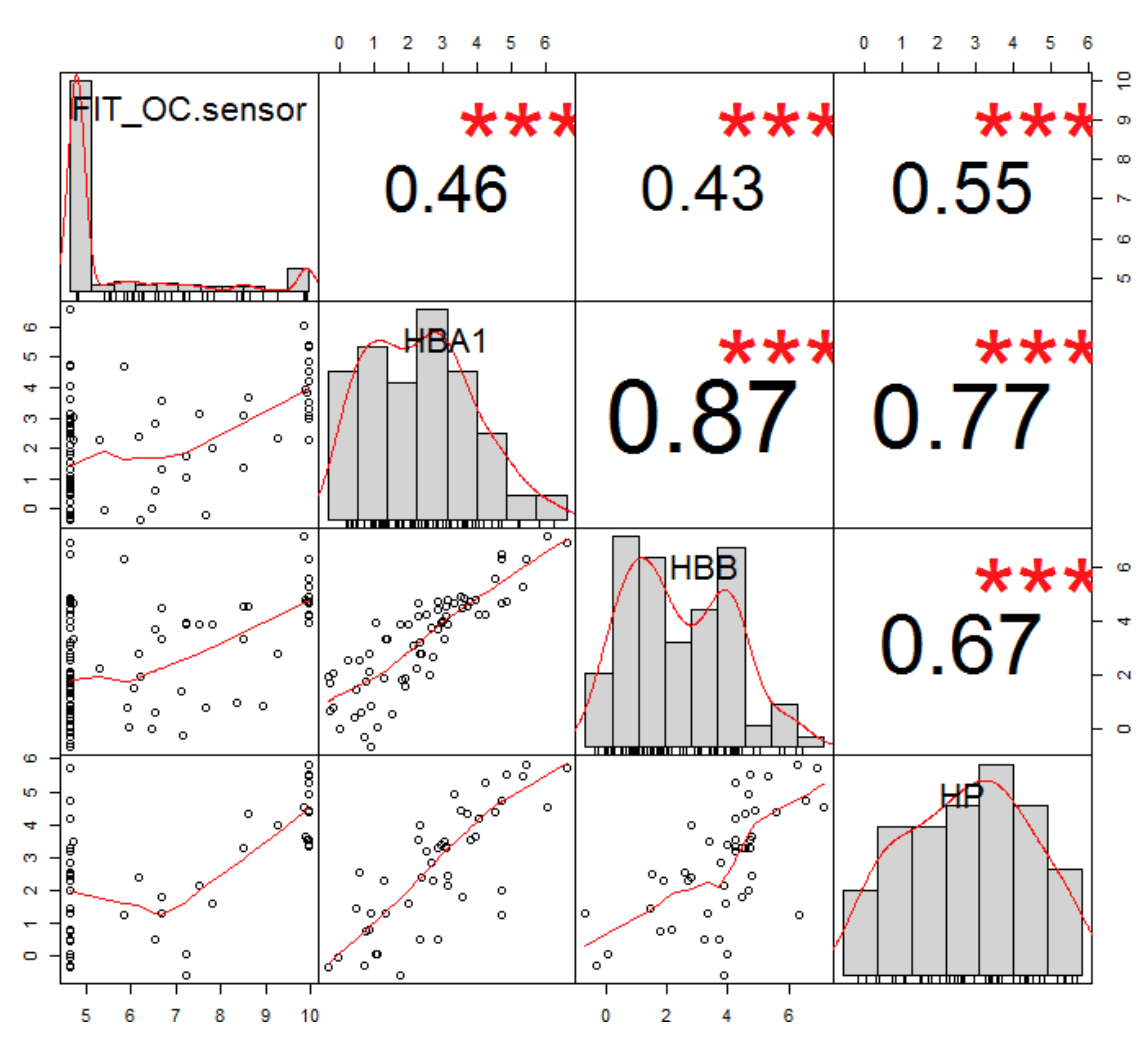


**Figure S3.** Spearman correlation analysis of hemoglobin (HBA1, HBB) and haptoglobin (HP) spectral counts and FIT values. Logarithmic transformation was applied on spectral counts and FIT values. The correlation analysis was performed on all the samples for which FIT values were available, including healthy controls (*n* = 96), low-risk adenomas (*n* = 43), high-risk adenomas (n = 10), unclassified adenomas (*n* = 8), and CRCs (*n* = 17). Bottom left matrix presents bivariate scatter plots with a fitted line. Top right displays correlation coefficient and significance level, where *** means *P* value ≤ 0.001.

**
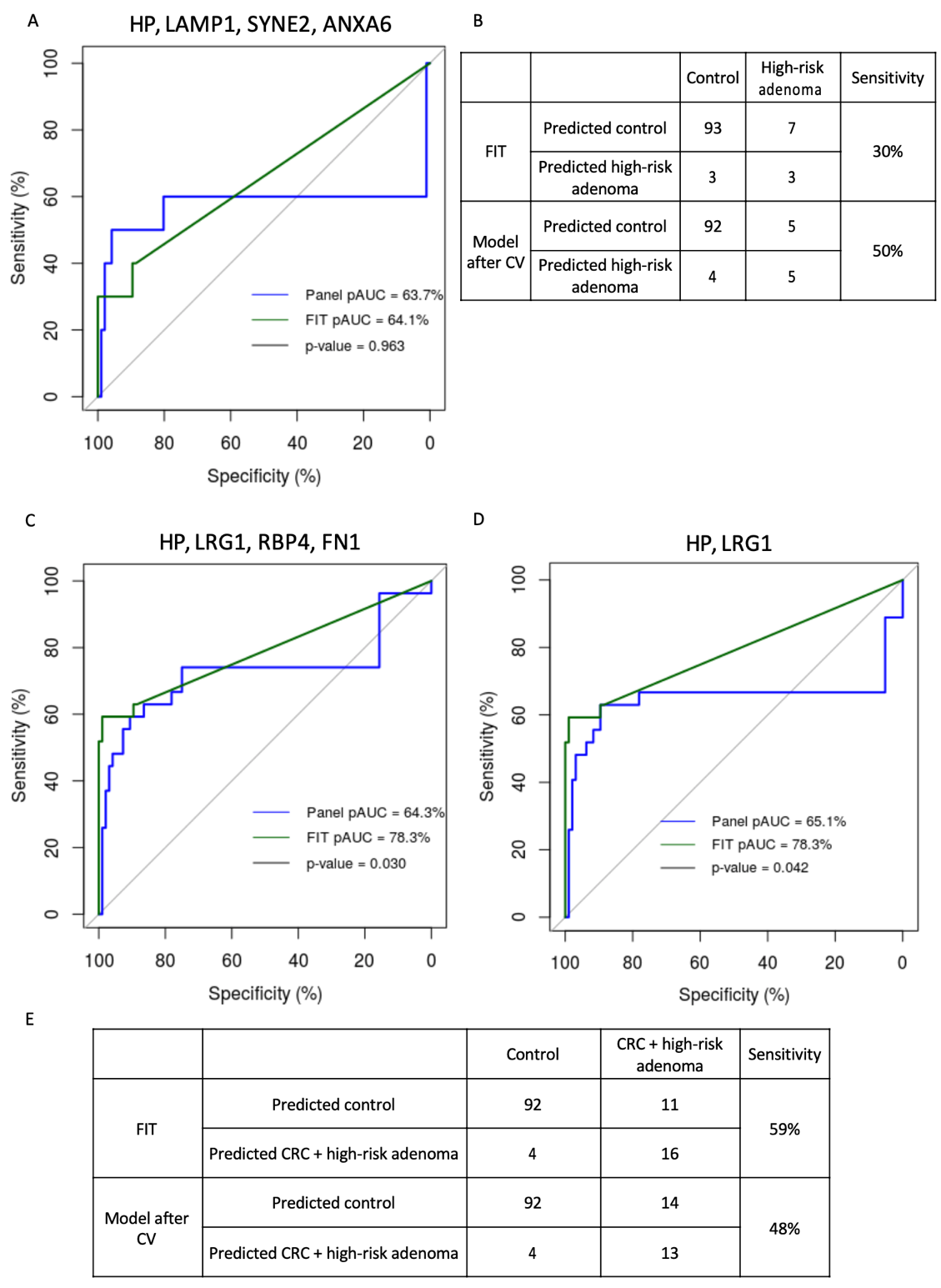
**

**Figure S4.** Comparison of the biomarker panels to FIT values. The FIT data were available for healthy controls (*n* = 96), high-risk adenomas (*n*= 10), and CRCs (*n*= 17). The cross-validated performance of the four-protein model was evaluated for high-risk adenoma identification (A, B); pAUC was calculated for ROC curve at the specificity level of 95–100% and compared with FIT values. Sensitivities of the model and FIT were evaluated at 95% specificity (B). For identification of high-risk adenomas and CRCs, four- (C) and two-feature (D) cross-validated models were evaluated with pAUC for ROC curves at the specificity level of 95–100%. Sensitivities for 95% specificity for both models resulted in the same sensitivity, which was compared with FIT (E).


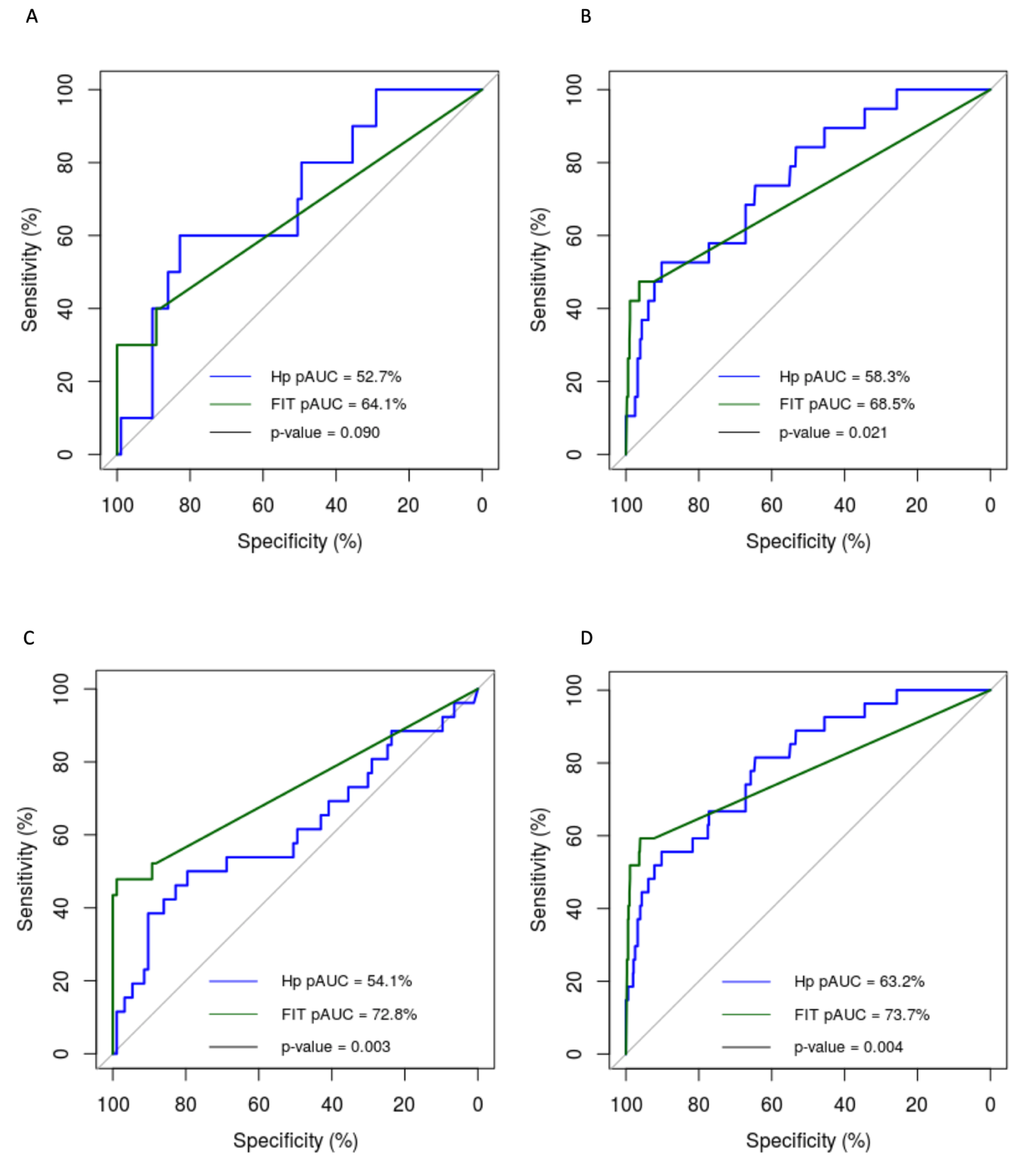


**Figure S5.** Comparison of the diagnostic performance of FIT and haptoglobin (Hp) measured with an antibody-based assay for high-risk adenomas (A, B) and high-risk adenomas with CRCs (C, D). ROC curves were obtained and pAUC at the specificity level of 95–100% was calculated separately for the study series (A, C) and the validation series (B, D). The study series consisted of 93 healthy controls, 10 high-risk adenomas, and 16 CRCs, while the validation series included 716 healthy controls, 19 high-risk adenomas, and 8 CRCs.
